# The Interaction of Biofoulants and Calcareous Deposits on Corrosion Performance of Q235 in Seawater

**DOI:** 10.3390/ma13040850

**Published:** 2020-02-13

**Authors:** Jie Zhang, Zhenhua Yu, Xia Zhao, Xiao Lan, Jiangwei Wang, Xianzi Lv, Chao Zhang, Jizhou Duan, Baorong Hou

**Affiliations:** 1 Key Laboratory of Marine Environmental Corrosion and Bio-Fouling, Institute of Oceanology, Chinese Academy of Sciences, Qingdao 266000, Chinasdqdjm188@163.com (J.W.); lvxianzi@qdio.ac.cn (X.L.); qiaoqiao8990@163.com (C.Z.); duanjz@qdio.ac.cn (J.D.); brhou@qdio.ac.cn (B.H.); 2 Open Studio for Marine Corrsion and Protection, Pilot National Laboratory for Marine Science and Technology, Qingdao 266000, China; 3 Center for Ocean Mega-Science, Chinese Academy of Sciences, Qingdao 266000, China; 4 Qingdao Municipal Center for Disease Control and Prevention, Qingdao 266000, China; yzhhgm607@163.com

**Keywords:** carbon steel, marine corrosion, calcareous deposit, cathodic protection, biofouling

## Abstract

An understanding of the interaction of calcareous deposits and biofoulants on the corrosion performance of steel during the fouling stage is both interesting and necessary. So, the effects of these factors on Q235 carbon steel were investigated and discussed for 20 weeks under real ocean conditions. The results indicate that calcareous deposits are favorable for the attachment of marine microorganisms. However, macroorganisms prefer adhering directly to the substrate. The generations of calcareous deposits have priority over the biofilm attachment under the condition of cathodic protection. Calcareous deposits can prevent steel against corrosion for four weeks without cathodic protection.

## 1. Introduction

Biofouling and seawater corrosion are two natural processes that occur at the interface between the metal and seawater, and they are important factors threatening the safety of sea-based facilities [1]. Biofouling on submerged surfaces in the marine environment has attracted particularly serious problems for shipping, platform, and coastal industries [2]. Natural seawater sources contain a diverse community of microorganisms and macroorganisms, including microbes, algae, invertebrate larvae, and other macroorganisms. The accumulation of microorganisms on the materials surface can also lead to microbially-induced corrosion (MIC), which has been extensively investigated for decades [3,4,5,6,7]. Macroorganisms interact with the biofilm and colonize on the surface [8,9], and the resultant growth of macroorganisms is a major industrial problem causing the corrosion of offshore structures [10,11]. The attachment of fouling organisms and their metabolites can modify the physicochemical properties of metal surfaces and create microenvironments, which can influence the corrosion process by changing the cathodic or anodic reactions [12,13,14,15,16].

With the development of marine engineering, cathodic protection, an efficient method of corrosion control, has been widely applied to marine structures, such as offshore platforms, subsea pipelines, as well as bridges and tunnels [17,18,19]. Calcareous deposits, as interesting secondary products in the cathodic protection process, have attracted widespread attentions in recent years because of their important roles in cathodic protection [20,21,22]. Lee [23] and Rossi [24] found that the calcareous deposits cover the structure surface and inhibit oxygen reduction, consequently decreasing the corrosion rate and improving the protective efficiency. This phenomenon is important in improving protection economics. In the decades since these studies, various parameters affecting the formation of calcareous deposits on marine structures have been investigated [25]. Some tests have been conducted, either in the laboratory or actual field, into applying cathodic protection to carbon steel to investigate the influence of marine sediments, clay, bacteria, and various ions on the formation of calcareous deposits [25,26,27,28].

The effect of biofouling on the sacrificial anodes used to control the corrosion and formation of calcareous deposits has also been reported. Blackwood et al. [29] investigated the influence of fouling on the efficiency of sacrificial anodes providing cathodic protection and argued that both aluminum and zinc sacrificial anodes remain effective even after complete coverage by biofouling. Eashwar et al. [30] examined the effects of biofilms on the cathodic behavior of stainless steel during galvanic coupling with anodic metal and determined that biofilms altered the cathodic current but were overwhelmed by calcareous deposits. Refait et al. [19] placed carbon steel in the tidal zone with and without cathodic protection and found that a thin biofouling film had formed on the steel surface under the calcareous deposits. In contrast, organic metabolites, such as acetic acid and fatty acids, can dissolve the protective calcareous deposits, which increase the polarizing current density and alter the Ca/Mg ratio [31]. The organisms’ fouling marine structures are affected by several factors, including location, hydrology, season, and the properties of the substrate [32,33].

However, previous investigations concerning the biofouling on calcareous deposits are mostly focused on single microfouling or macrofouling, which are also short of in-depth exploration. The reports concerning the protective effects of calcareous deposits on the corrosion performance of metallic materials over the entire fouling process have not yet been totally explored, which involve microorganism and macroorganism attachment. More importantly, the corrosion rates and foul in actual marine environment are very much location-specific, and some laboratory studies frequently fail to reproduce the actual rates found in the field [34]. So, it is important to conduct the research in an actual marine environment, which can provide the real environmental parameters and simulate the entire fouling stage, including the easy micro-fouling stages and the late macro-fouling stages.

In the present study, a 20-week test was conducted in a real ocean environment to investigate the corrosion resistance of calcareous deposits formed by a sacrificial anode on Q235 carbon steel and any changes induced by the attachment of biofoulants during the fouling stage. The relationships between the biofouling process, calcareous deposits, and corrosion behavior of carbon steel under cathodic protection were also investigated. The interactions between microbes, algae, and macroorganism with calcareous deposits were also analyzed in detail.

## 2. Materials and Methods

### 2.1. Experimental Conditions

Experiments were performed in a port in Jiaozhou Bay, China for 20 weeks. Authoritative data indicated that the sea surface temperature varied between 20 and 25 °C, the dissolved oxygen value was 8.4 mg·L^−1^, the salinity value was 32, the pH value was 8.3, and the flow velocity was low. Representative fouling organisms encountered during the experimental period included Calcarina, moss nematodes, barnacles, oysters, mussels, and Ciona.

### 2.2. The Chemical Composition of Q235

**Figure 1:**
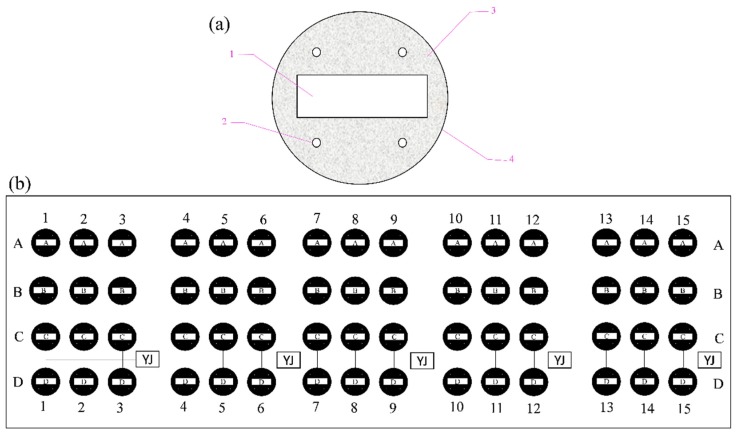
Experimental samples and fixed frame. (**a**) Carbon steel electrodes: 1, Q235 carbon steel; 2, polymeric tubes; 3, nonconducting epoxy resin; 4, mold. (**b**) Experimental polyethylene frame: anode-uncoupled bare steel samples (row of samples labeled A); anode-coupled steel samples without pre-existing calcareous deposits (row of samples labeled B); anode-uncoupled steel samples with pre-existing calcareous deposits (row of samples labeled C); and anode-coupled steel samples with pre-existing calcareous deposits (row of samples labeled D).

The Q235 carbon steel panel used in the experiment consisted of (wt %) 0.1, C; 0.4, Mn; 0.12, Si; 0.02, S; 0.05, P; and Fe balance. The steel sample was cut into a rectangular block (20 mm × 50 mm × 5 mm) and embedded in a mold (Ø70 × 10 mm) with nonconducting epoxy resin. The resultant sample was attached to a copper conductor (with a diameter of 0.2 mm) by welding, leaving only one main section of the carbon steel exposed. Additionally, four polymeric tubes (Ø10 mm× 12 mm) were appropriately embedded in epoxy around each sample (Figure 1a). Following prior work, the exposed surface was ground using 800- and 1200-grit emery paper. To investigate the biofouling attachment and corrosion resistance of pure calcareous deposits, calcareous deposits were formed on 30 samples by cathodic polarization. A constant current of −300 μA was impressed on one sample immersed in natural seawater for 72 h. In this system, a saturated calomel electrode (SCE) and graphite plate served as a reference electrode and counter electrode, respectively. Then, 30 samples with pre-existing calcareous deposits were stored in a desiccator for the subsequent investigation.

The established electrodes were installed on a polyethylene frame with 240 evenly distributed holes (Ø10 mm) attached to polymeric tubes for mounting to the electrodes by self-locking rack binders. Sixty carbon steel electrodes were present in the whole panel and were divided into four categories as follows: anode-uncoupled bare steel samples (without pre-existing calcareous deposits, schematically illustrated in row A in Figure 1b), anode-coupled steel samples without pre-existing calcareous deposits (with sacrificial anode protection, schematically illustrated in row B in Figure 1b), anode-uncoupled steel samples with pre-existing calcareous deposits (without sacrificial anode protection, schematically illustrated in row C in Figure 1b), and anode-coupled steel samples with pre-existing calcareous deposits (with sacrificial anode protection, schematically illustrated in row D in Figure 1b). Then, all junctions were sealed with insulating rubber tape and epoxy resin.

When we arrived at the port, the anode-coupled steel samples (both bare and those with pre-existing calcareous deposits) have been connected through cathodic protection by galvanic coupling with a Zn-Al-Cd alloy sacrificial anode before being put into the seawater. The surface area ratio of the cathode to the anode was 10:1, as recommended by an engineering manual.

The samples were exposed for 1 week (schematically illustrated in columns 1–3 in Figure 1b), 2 weeks (schematically illustrated in columns 4–6 in Figure 1b), 4 weeks (schematically illustrated in columns 7–9 in Figure 1b), 8 weeks (schematically illustrated in columns 10–12 in Figure 1b), and 20 weeks (schematically illustrated in columns 13–15 in Figure 1b) respectively.

The frame was hung in a floating raft and immersed in seawater on the sunward side, reaching a depth of 1 m. The electrode potential of the galvanic coupling against SCE was frequently monitored using a multimeter (AVOmeter). The frame was collected, and then some steel panels were sampled and returned to the laboratory for subsequent tests after exposure in seawater for 1, 2, 4, 8, and 20 weeks respectively.

### 2.3. Electrochemical Procedure and Measurements

After sampling from the real ocean environment, each electrochemical measurement was conducted within 2 h. A specialized cell structure was designed to measure the electrical parameters. A stepped hole (Ø10 mm) was left on the flank, so the actual exposed area of the working electrode used in the electrochemical procedure was 78.5 mm^2^ (Ø10 mm). The counter and reference electrodes comprised platinum and SCE, respectively. Fresh, filtered seawater was used as the electrolyte in the electrochemical measurements.

For polarization resistance determination, the linear potential was measured at a potential scanning rate of 0.5 mV/s from the open circuit potential (*E*_OCP_) − 250 mV to *E*_OCP_ + 250 mV until the *E*_OCP_ of the working electrode attained a steady state [35]. The linear potential was scanned at the rate of 0.5 mV·s^−1^ from the corrosion potential (*E*c, mV) to ±10 mV relative to *E*c. Electrochemical impedance spectroscopy (EIS) measurements were conducted at the OCP by applying a sinusoidal voltage of 10 mV (versus the SCE) from 100 kHz to 10 mHz at 7 points per decade. The potentiodynamic polarization curves and EIS measurements were performed with a Solartron SI1287 electrochemical interface and SI1260 impedance/gain-phase analyzer (Solartron, London, UK), respectively. The data for the polarization Tafel curves were fitted using the CView2 software.

### 2.4. Observation of Fouling Organisms by Fluorescence Microscopy

The attachment of microorganisms in the early period of the experiments was examined by fluorescence microscopy. The immersed samples were used to investigate the attachment of biofoulants in a 5% glutaraldehyde phosphate buffer for 15 min after thorough cleaning with phosphate buffer. Then, the samples were stained with a 0.2% fluorescent stain (4,6-diamidino-2-phenylindole) for 10 min under dark and aseptic conditions.

### 2.5. SEM Observations of Microorganisms

The surfaces of the samples immersed in the early phase of the experiments (one and two weeks) were observed by scanning electron microscopy (SEM,S-4300, Hitach, Tokyo, Japan) and analyzed by energy-dispersive X-ray spectroscopy (EDS, S-4300, Hitach, Tokyo, Japan). The samples were prepared by the following process. Sea sediments were removed and pretreated by dehydration in a graded ethanol series. Then, the organisms were fixed with a 5% glutaraldehyde phosphate buffer. The samples were finally subjected to carbon dioxide critical point drying and sputtered with gold to allow SEM and EDS measurements.

### 2.6. SEM Analysis of the Corrosion Morphology

SEM was used to observe the corrosion morphology under the calcareous deposits or corrosion products following long-term exposure (one, two, four, eight, and 20 weeks). The morphology of the steel samples after corrosion was also observed by SEM. To observe the corrosion morphology, the samples were cleaned according to GB/T 16545-2015 (Chinese National Standard). First, the organisms attached to the exposed surface were removed with a bristle brush, and the samples were immersed into an acid cleaning solution (1 L 37% HCl, 20 g Sb_2_O_3_, 50 g SnCl_2_) for 10 min, for which smooth rubbing was necessary. Then, these samples were rinsed with distilled water and alkaline solution (0.1 M NaOH) and cleansed successively with distilled water, followed by pure ethanol again, and dried under nitrogen flow.

## 3. Results and Discussion

### 3.1. Biofouling Process

**Figure 2:**
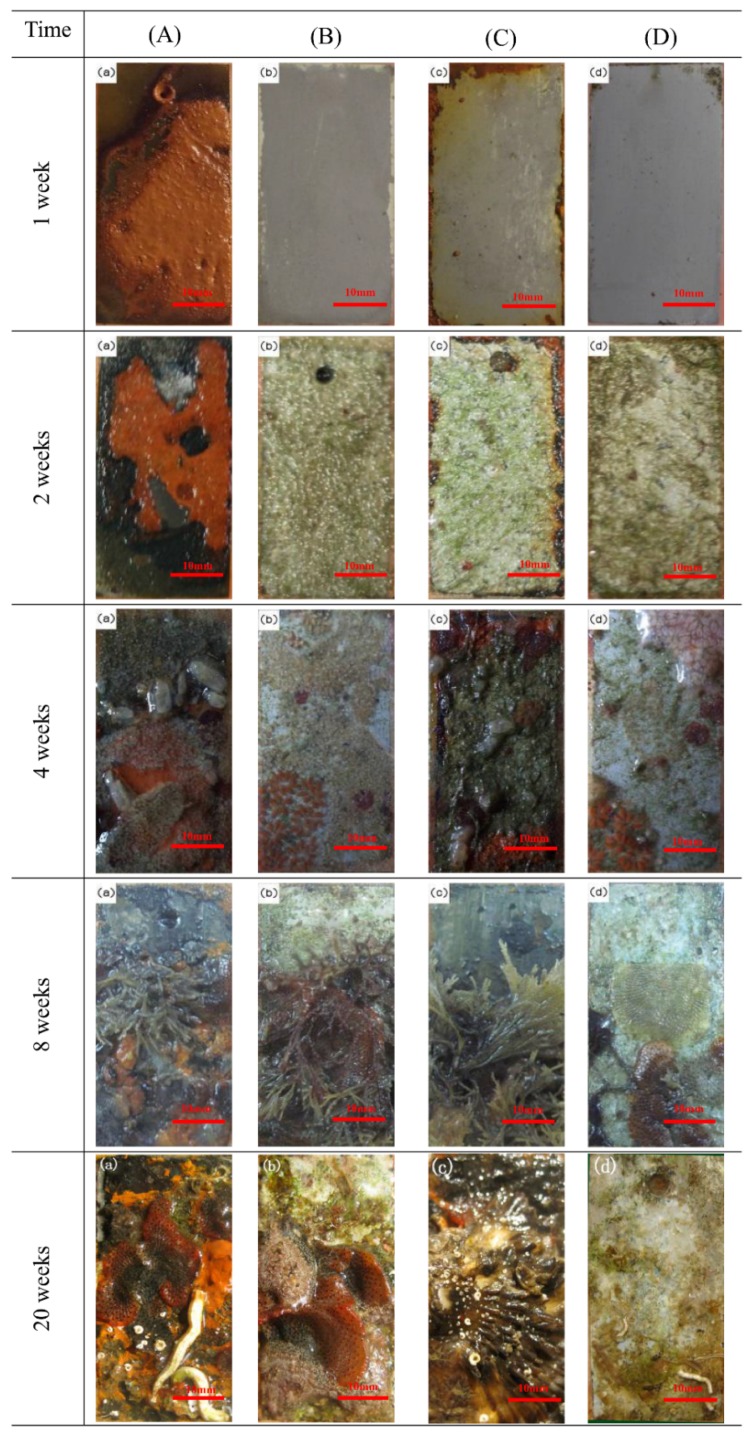
Digital images of samples under different conditions. (**A**) Anode-uncoupled bare samples; (**B**) anode-coupled bare samples; (**C**) anode-uncoupled samples with pre-existing calcareous deposits; (**D**) anode-coupled samples with pre-existing calcareous deposits.

The changes to the specimens throughout the exposure tests were recorded using a digital camera (Figure 2). Visual inspection revealed that the calcareous deposits completely covered the anode-coupled steel samples (Figure 2B,D), and no defects were observed around the edges after the first week. However, significant corrosion occurred on the unprotected counterparts (Figure 2A), which were covered in the double-oxide product with a loose and unsteady outer layer and strongly attached inner layer. The unprotected samples with pre-existing calcareous deposits were subjected to slight corrosion (Figure 2C), but serious corrosion occurred for the anode-uncoupled bare steel samples, which implies that the calcareous deposits offered certain protection to the carbon steel submerged in the sea for four weeks.

In the early phase of the experiment, there was no noticeable colonization of organisms. For all of the specimens, a visible, clear biofouling film covered of the entire surface after four weeks. Previous research indicates that this biofouling film consists of larvae and spores, soft-bodied organisms, and metabolites [36]. Furthermore, sessile organisms replaced moss nematodes after eight-week exposure, whereas the previously mentioned biofouling film and their metabolites decayed and decomposed in this phase. After 20 weeks, a few representative fouling organisms (Ciona intestinalis, barnacles, oysters, and mussels) began to thrive on the outer layer (Figure of 20 weeks (a), (b), (c) in Figure 2). During this period, the number of organisms attached to the protected specimen was less than those of the unprotected counterparts.

**Figure 3:**
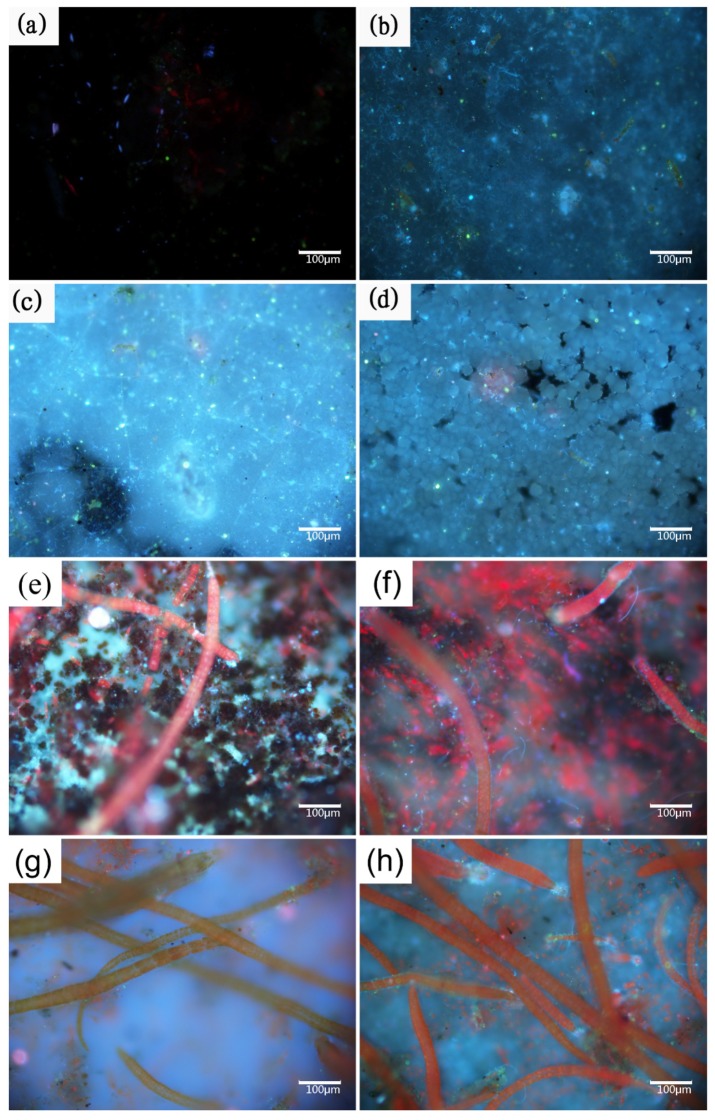
Fluorescence images of: anode-uncoupled bare samples (((**a**), 1 week), ((**e**), 2 weeks)), anode-coupled bare samples (((**b**), 1 week) ((**f**), 2 weeks)), anode-uncoupled samples with pre-existing calcareous deposits (((**c**), 1 week) ((**g**), 2 weeks)), and anode-coupled samples with pre-existing calcareous deposits (((**d**), 1 week), ((**h**), 2 weeks)), all immersed in natural seawater.

In previous reports, the fouling organisms attached to the specimens during the early phase of the experiments were microorganisms: bacteria and microalgae for about one week [25]; then, there was algae for about two weeks, followed by spores and larvae about four weeks. Obtaining precise counts of the biofilm microorganisms attached to each specimen is difficult because of the many uncultured bacteria in the marine environment [37]. In our paper, the conditions of the microorganisms attached to the central region of each specimen were characterized by fluorescence microscopy, which can reveal much useful information, as shown in Figure 3. Stained bacteria and algae emit blue and red fluorescent light, respectively, when stimulated by 510–550 nm (ultraviolet) light. Samples with calcareous deposits were easily colonized by microorganisms [38,39], which is consistent with the results shown in Figure 2, and the biofouling adhesion was progressive. Therefore, the bacterial biofilm, which is frequently associated with algae [40], grew on submerged structures in the marine environment (two weeks in Figure 2). Concerning the spores and larvae of fouling organisms, a biofouling film-covered surface was a prerequisite for settlement. Moreover, the attachment of macroorganisms always began from their spores and larvae. Therefore, colonization by biofouling successively alternated from bacterial film to macroorganisms [41]. The properties of the former were expected to either positively or negatively influence the colonization of the latter. The factors affecting the colonization of biofoulants include the temperature, pH, salinity, flow velocity, nature of the material, and illumination.

In our case, the specimens were covered by biofilm composed of bacteria, algae, and metabolites during early exposure (one and two weeks, Figure 3a–h). The specimens with pre-existing calcareous deposits are typical for more susceptible to biofouling. Fouling organisms attach themselves to the substrate by secreting bioadhesives consisting of proteins, polysaccharides, polyphenols, and lipids [42]. The adsorption energies of calcium carbonate, the main component of the calcareous deposits, are typical chemisorption for hydroxyl functional groups (-OH), resulting in the strong adsorption of bioadhesives [43]. In the case of cellular processes, calcium ions play an essential role in the adhesion of cells to surfaces and participate in nonspecific interactions, such as the neutralization of the electrical double layer between the cell and substratum surface, as well as specific adhesive interactions [44]. The properties of calcium ions promote both specific and nonspecific interactions with the protein and polysaccharide adhesion molecules at the cell surface. However, studying biofouling under cathodic polarization is challenging. Most studies have focused on microorganisms, but a few authors have reported that more bacterial settlements are observed on the cathodically protected materials, which agrees with our test results [45]. However, the opposite view has also been reported, where there is no variation in bacterial fouling and the metabolic activity is observed to decrease in both bacteria and marine diatoms [46]. Few authors have attributed this discrepancy to a difference in experimental conditions, such as between the field and laboratory [47].

For macroorganisms, the experiment results showed that macrofoulants adhered less strongly to the surface with existing calcareous deposits than to the bare surface, especially for the anode-coupled samples with pre-existing calcareous deposits, where there was almost no macrofoulants attachment. Other researchers have come to the same conclusion [29,30]. The reason may be that these macroorganisms easily suffered from scouring by flow, and the barnacles and oysters adhering in the former were removed more easily with deposits under the scouring of flow. Accordingly, the specimens were exposed and preferred to attach to bare steel surfaces. Other reports considered that the adhesion of macrofouling is adversely affected by a high alkalinity in the local environment and cathodic polarization, which reduces metabolic activity, leading to decreased adhesion strengths [29].

### 3.2. Calcareous Deposits Formation

**Figure 4:**
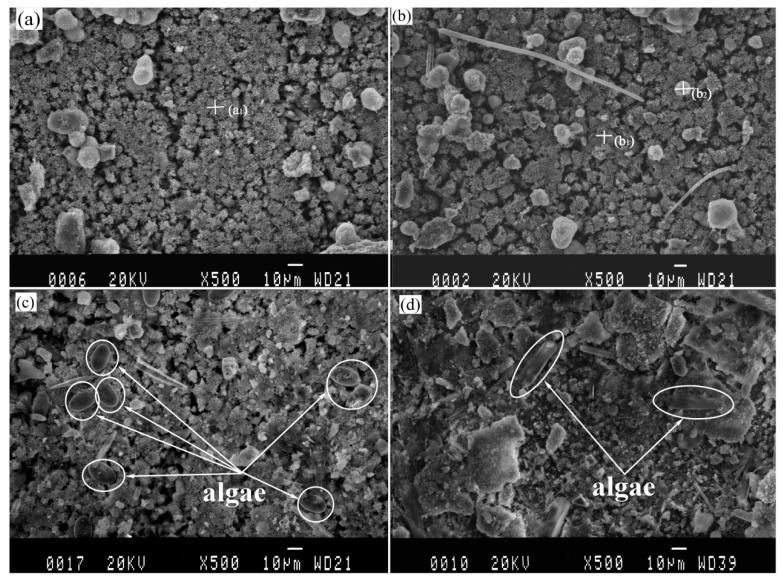
Images of calcareous deposits formed on anode-coupled bare samples (((**a**), 1 week), ((**c**), 2 week)) and anode-coupled samples with pre-existing calcareous deposits (((**b**), 1 week), ((**d**), 2 week)) after exposure in natural seawater.

The SEM images analysis of protected specimens are shown in Figure 4. The samples are similar in terms of elemental composition and morphology, regardless of the pre-existing calcareous deposit or not, which is considered as new deposits generated continually on the surface of pre-existing deposits under the cathodic protection. In order to study the differences between the calcareous deposits prepared using cathodic polarization in laboratory and that prepared by connecting anode in actual marine environment, EDS analysis was considered. The EDS results of the new calcareous deposits formed on the anode-coupled bare steel samples and the anode-coupled steel samples with pre-existing deposits after exposure for one week to natural seawater are shown in Table 1. The chemical composition of the deposits was thought to be CaCO_3_, based on the detected main elements (Ca, O, and C) in agreement with our previous XRD results [48]. A small amount of Mg(OH)_2_ was mixed with this compound. A few spherical grains were dispersed on the surface of the deposits (see in Figure 4), originating from clay suspended in seawater. However, the microbes are so small (about 1 μm) that they cannot be determined in Figure 4a,b, but the microbes can be distinguished exactly in Figure 3a–d. Observation of the samples immersed for two weeks revealed clay particles and fragments of organisms covering the whole surface of the deposits (see in Figure 4). Algae would be observed in Figure 4c,d, but it was obviously different that the Algae in Figure 4c, which could be Coscinodiscus, while in Figure 4d, it could be Navicula. For the anode-coupled bare samples submerged in the sea for two weeks, the surface was a non-stationary state of polarization, and the cathodic protection potential had not yet reached stabilization, so the calcareous deposits formed on these samples were not yet fully formed. Compared Figure 4c,d, we thought that Coscinodiscus prefer to adhere directly to the substrate, while Navicula prefer to adhere to the calcareous deposits. The reason is still unknown because of the complexity of the algae, which needs further research in the future.

This result is consistent with the fluorescence images (Figure 3a,c) and implies that the generations of calcareous deposits have priority over the biofilm attachment under cathodic protection. Although the surfaces of the specimens protected by pre-existing deposits were covered with biofilms after one week of exposure, calcareous deposits can build up below the biofilm.

Calcareous deposits formed on cathodically protected samples suffering from the influence of biofouling and marine sediment in the field [49]. Marine sediments contain a wide variety of organic and inorganic components that reduce the calcareous deposition kinetics and increase the final current [49]. A few reports have attributed these factors to the reduction of the active surface [49]. A reverse test found that whether or not clay particles exist has little effect on the deposition kinetics of calcareous deposits [25]. Calcareous deposits initially formed directly on the sediment particles and covered them entirely, and then the calcareous deposits with sediment particles connected together. This growth has been observed under a microscope, and it was shown as a polygon with dispersed particles (Figure 4a,b), which is consistent with the results of other researchers [25]. The similarity of chemical components in the deposits formed on the bare steel sample and on the pre-existing deposits may be ascribed to calcareous deposits growing on the pre-existing deposits, confirming the above-mentioned mechanism.

### 3.3. Electrochemical Analysis

**Figure 5:**
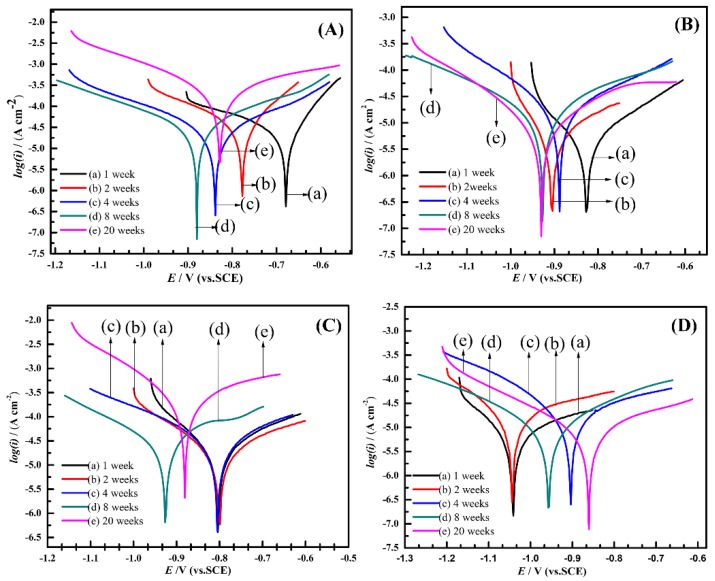
Polarization curves for anode-uncoupled bare samples (**A**), anode-coupled bare samples (**B**), anode-uncoupled samples with pre-existing calcareous deposits (**C**), and anode-coupled samples with pre-existing calcareous deposits (**D**), all exposed to seawater for various periods.

All electrochemical measurements were conducted in filtered seawater. Each sample was measured three times in different regions to acquire parallel data. Figure 5 illustrates the polarization characteristics of the specimens exposed to natural seawater under various conditions. The polarization behavior of the anode-uncoupled bare steel samples over time is shown in Figure 5A. Within the first eight weeks, the corrosion potential became negative, and the current density increased slightly; after 20 weeks, the corrosion potential became positive and the corrosion current density increased significantly, which showed that the samples occurred serious corrosion. A series of semilogarithmic polarization curves show the results of electrochemical measurements of the anode-coupled bare steel samples (Figure 5B); these curves show a relatively steady trend; namely, the potentials regularly turned negative and ranged in the region of 0.1 V. The corrosion potentials reached −926 mV (versus SCE) after eight weeks and then remained stable, so we can conclude that the samples reached a stable cathodic protection potential over this period. For the unprotected samples with pre-existing deposits, the potential remained stable over the first four weeks, which may be related to the protection afforded by the pre-existing deposits on the samples. Subsequently, the corrosion potential became noticeably negative after immersion for eight weeks, and the current density increased considerably over 20 weeks (Figure 5C); the reason for this behavior is considered to be the pre-existing deposits losing its protective effect after four weeks in a real ocean environment. The curves of the protected samples with pre-existing deposits (Figure 5D) indicate that the corrosion potentials reached a value of −1042 mV (versus the SCE) at the beginning of the exposure experiment, which shows that the samples could easily reach a stable cathodic protection potential with the protection of the pre-existing calcareous deposits.

To characterize the effect of the microorganisms on the corrosion rate, the corrosion current density (i_corr_) was calculated using the Stern–Geary equation [50,51,52], as shown in Equation (1).

i_corr_ = (b_c_·b_a_)/(2.303·R_p_(b_c_ + b_a_)), (1)

**Figure 6:**
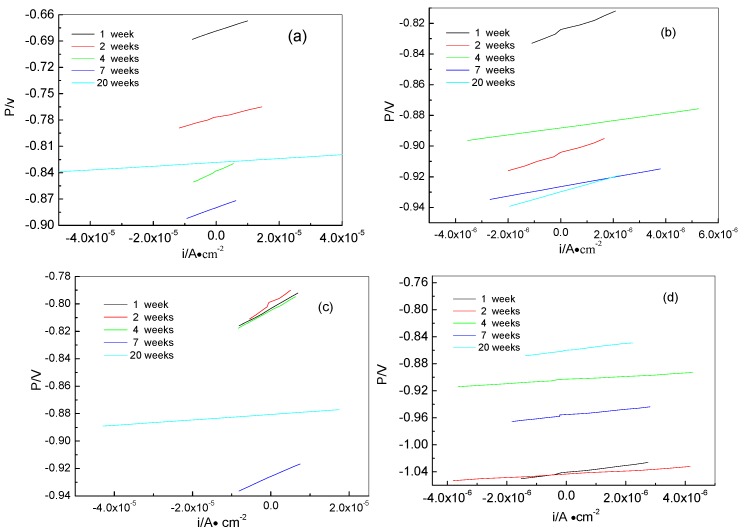
Liner polarization curves of samples for different time: anode-uncoupled bare samples (**a**), anode-coupled bare samples (**b**), anode-uncoupled samples with pre-existing calcareous deposits (**c**), and anode-coupled samples with pre-existing calcareous deposits (**d**).

Here, b_a_ and b_c_ are the anodic and cathodic Tafel slopes respectively, which can be obtained from the polarization curves in Figure 5. R_p_ is the polarization resistance, which can be calculated from the linear potential curve slope with the current on the *x*-axis and the potential on the *y*-axis. The linear polarization curves of samples for different times are shown in Figure 6. All corrosion parameters are listed in Table 2. From Table 2, we can see that the i_corr_ values of the anode-uncoupled bare steel samples are much larger than those of the anode-coupled bare steel samples during the experimental exposure, which shows that sacrificial-anode cathodic protection can protect samples against marine corrosion.

Comparison of the anode-coupled steel samples (B and D in Table 2) shows that the E_ocp_ values of the anode-coupled samples with pre-existing calcareous deposits are more negative than that of samples without pre-existing calcareous deposits after immersion for two weeks, which implies that the surface of the anode-coupled bare samples is a non-stationary state of polarization, and the cathodic protection potential has not yet reached stabilization.

Comparing the anode-uncoupled bare steel samples and the anode-uncoupled steel samples with pre-existing calcareous deposits, these data show that the corrosion rate of the anode-uncoupled bare steel samples is increased from the first week to the second week, which implies that the vigorous metabolic activities of biofouling promote the occurrence of corrosion. However, the corrosion rate of the anode-uncoupled steel samples with pre-existing calcareous deposits is stable, which shows that the influence of biofouling attachment is smaller than that of calcareous deposits on the corrosion rate of samples. Within the first four weeks, the pre-existing calcareous deposits offered better protection for the Q235 steel, but this protective effect was lost after eight weeks, as shown by the similar i_corr_ values of the anode-uncoupled steel samples with pre-existing calcareous deposits to those of the anode-uncoupled bare steel samples. This is also consistent with the results shown in Figure 2.

Table 2 also shows that for the anode-uncoupled steel samples, both with pre-existing deposits or bare, the E_corr_ values were more positive than those of anode-coupled steel samples after 20 weeks, and the i_corr_ values of the anode-uncoupled steel samples are about 20 times larger than those of the anode-coupled steel samples. It is evident that the steel samples without cathodic protection suffered severe corrosion, both those with pre-existing deposits and the bare carbon steel samples in real ocean conditions.

**Figure 7:**
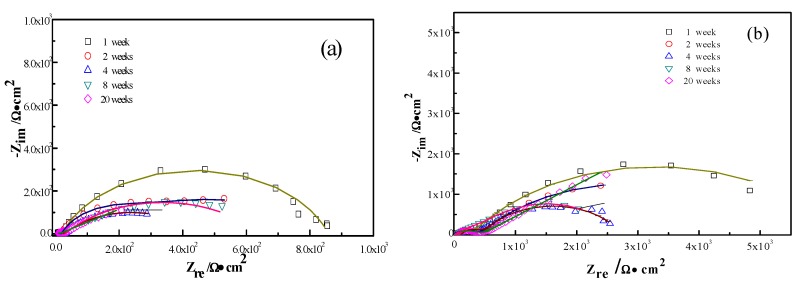

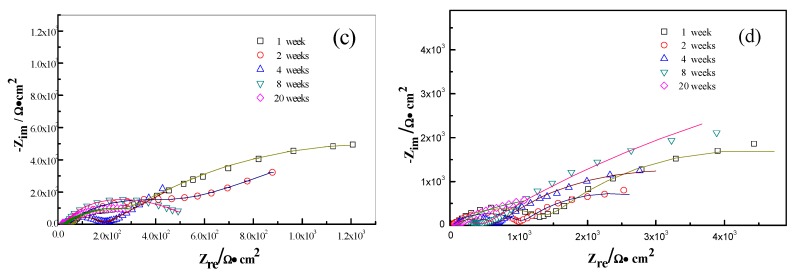
Nyquist plots of the measured (symbols) and fitted data (lines) for anode-uncoupled bare samples (**a**), anode-coupled bare samples (**b**), anode-uncoupled samples with pre-existing calcareous deposits (**c**), and anode-coupled samples with pre-existing calcareous deposits (**d**), all exposed to seawater for various periods.

**Figure 8:**
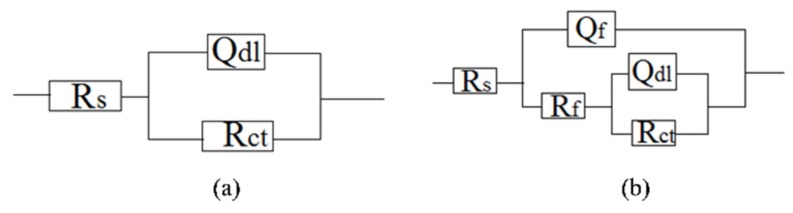
Equivalent circuits for (**a**) anode-uncoupled bare samples and (**b**) three other kinds of samples.

The interactions between the calcareous deposits, biofouling, and the corrosion of carbon steel were investigated by using EIS measurements in order to study the corrosion evolution in time. The EIS measurements were used to illustrate the mechanisms by which biofouling and calcareous deposition influence the corrosion of the steel. Figure 7 shows the Nyquist plots for carbon steel under various conditions. Two equivalent circuits were used to model the corrosion process of the electrode in similar conditions (Figure 8a for anode-uncoupled bare carbon steel and Figure 8b for three other samples). In these figures, R_s_ is the solution resistance, Q_f_ is the capacitance of the surface film, R_f_ is the resistance of the surface film, Q_dl_ is the capacitance of the double layer, and R_ct_ is the charge-transfer resistance (polarization resistance). Figure 7a shows the plots obtained for the anode-uncoupled bare steel samples during immersion for different periods. As shown in the figures, only one apparent semicircular loop and a single time constant were observed, implying a single layer corresponding to the interfacial process. A sharp decrease of semicircle diameter was observed in the Nyquist plots in the samples exposed for 1 and 2 weeks. During this phase, algae would become the dominant fouling agent and would produce adequate oxides in the interface between metal and seawater. This phenomenon induced the depolarization of oxygen cathodic reduction and increased the corrosion rate.

For the anode-coupled bare steel samples (Figure 7b) exposed for each period, the Nyquist plots reveal a semicircle in the low-frequency region and a small semicircle in the high-frequency region. The former is associated with the charge-transfer resistance (R_ct_) and capacitance of the double layer (Q_dl_), reflecting the interfacial electric double layer. The latter is associated with the film resistance (Rf) and film capacitance (Q_f_), reflecting the performance of the biofilm, biofouling film, and corrosion products. The successive decrease in the impedance arc diameter within the first eight weeks implies that the corrosion rate increased gradually, and the barrier efficiency of the calcareous deposits decreased. In contrast, the impedance of the arc diameter increased again after 20 weeks, which may be attributed to the accumulation of corrosive products under the calcareous deposits.

Figure 7c shows the impedance plots of anode-uncoupled steel samples with pre-existing calcareous deposits. There is a distinct difference between the curves in the first four-week exposure and the remaining periods. Therefore, the interfacial process changed, and, moreover, the protection efficiency of the pure calcareous deposits failed. Unlike the anode-uncoupled bare steel samples, the maximum impedance arc diameter was obtained after two-week exposure, which may be attributed to the formation of a dense biofilm layer on the calcareous deposits [19]. Consistent with anode-uncoupled bare steel samples, the impedance arc diameter remained small with the development of macroorganisms after eight-week exposure. However, the presence or absence of calcareous deposits induced changes. Therefore, the calcareous deposits inhibited the corrosion of the substrate submerged in the sea. However, the attachment of biofoulants had the opposite effect.

The anode-coupled steel samples with pre-existing calcareous deposits had efficient corrosion resistance. The impedance arc diameter from polarization curves was always larger than that of the anode-coupled bare steel samples before eight-week exposure (Figure 7d). Therefore, the pre-existing calcareous deposits accelerated the attachment of biofoulants. Moreover, the calcareous deposits and the inorganic/organic composite biofouling film mutually compensated for the defects, providing effective prevention against oxidizing substances.

### 3.4. Corrosion Evolution

**Figure 9:**
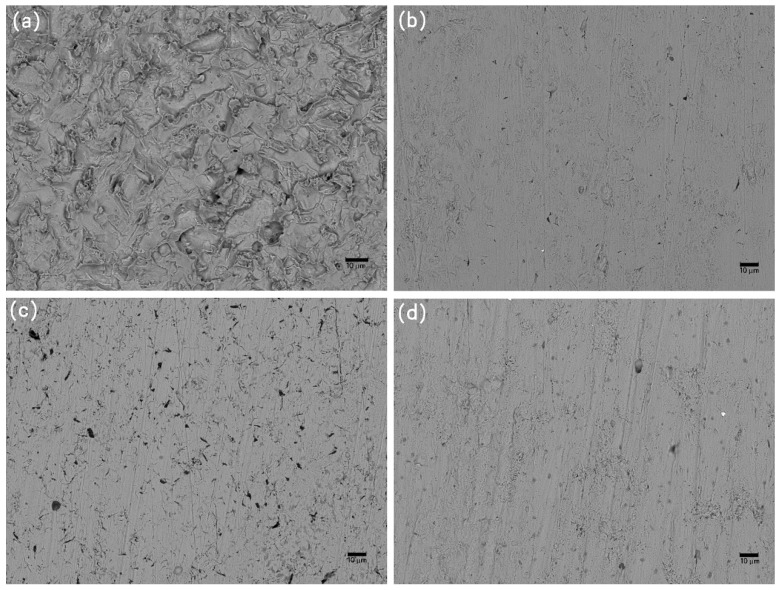
SEM images of corrosion morphology of anode-uncoupled bare samples (**a**), anode-coupled bare samples (**b**), anode-uncoupled samples with pre-existing calcareous deposits (**c**), and anode-coupled samples with pre-existing calcareous deposits (**d**), all exposed to seawater for 4 weeks.

**Figure 10:**
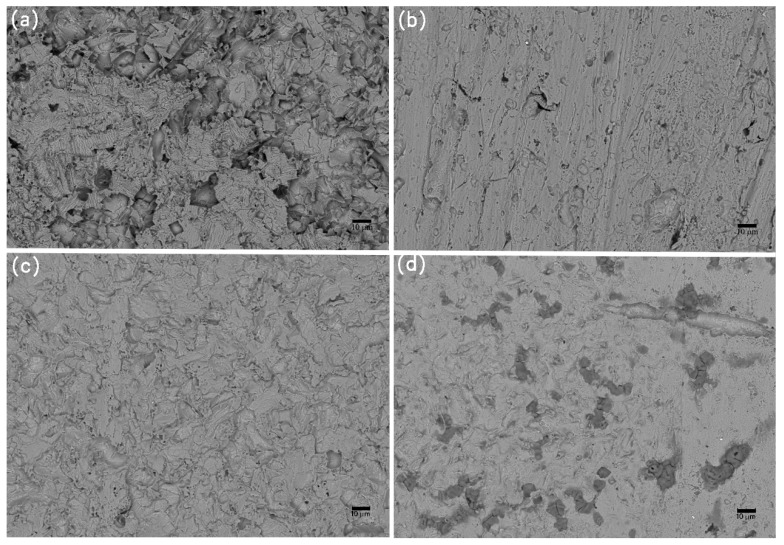
SEM images of corrosion morphology of anode-uncoupled bare samples (**a**), anode-coupled bare samples (**b**), anode-uncoupled samples with pre-existing calcareous deposits (**c**), and anode-coupled samples with pre-existing calcareous deposits (**d**), all exposed to seawater for 8 weeks.

**Figure 11:**
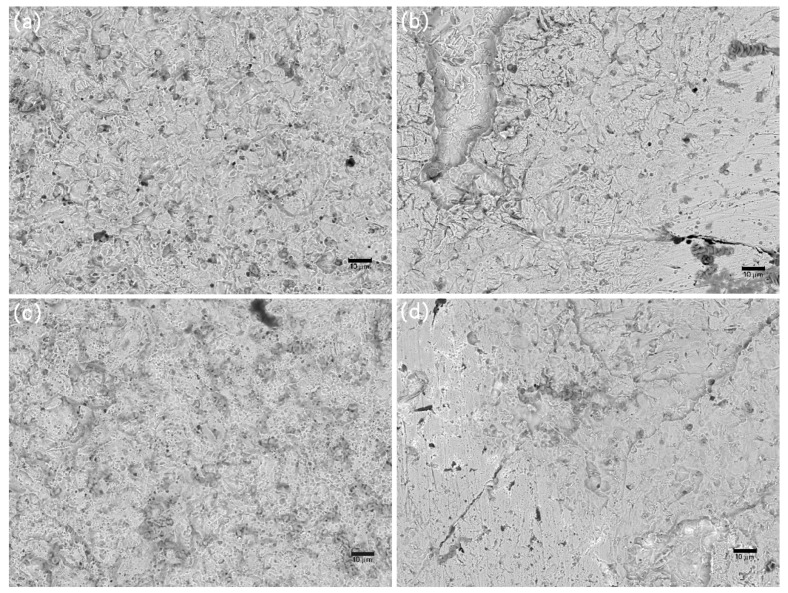
SEM images of corrosion morphology of anode-uncoupled bare samples (**a**), anode-coupled bare samples (**b**), anode-uncoupled samples with pre-existing calcareous deposits (**c**), and anode-coupled samples with pre-existing calcareous deposits (**d**), all exposed to seawater for 20 weeks.

The characteristic morphologies of the corroded samples, as analyzed using SEM after the removal of the foulants and products, are shown in Figure 9, Figure 10 and Figure 11. The galvanic corrosion formed when the macroorganisms began to colonize on local surface of the samples; afterwards, the localized corrosion pit would occur on the surface. After exposure for eight weeks, the corrosion became significant. This observation is consistent with the failure of the protective effect of the calcareous deposits. After 20 weeks, uniform corrosion occurred because the colonization of macroorganisms was dense. In fact, the corrosion degree for the samples without cathodic protection at the 8th week was lower than the samples without cathodic protection at the 20th week. The corrosion process for samples was changed from local corrosion to uniform corrosion.

## 4. Conclusions

The calcareous deposits obtained by cathodic protection are favorable for the attachment of marine microorganisms. The reason may be that the adsorption energies of calcium carbonate for hydroxyl functional groups are typical of chemisorption, resulting in the strong adsorption of bioadhesives. The properties of calcium ions promote both specific and nonspecific interactions with the protein and polysaccharide adhesion molecules at the cell surface.

Macroorganisms prefer to adhere directly to the substrate instead of the calcareous deposits, because macroorganisms easily suffer from scouring by flow and can be removed easily with deposits under the scouring of flow.

The generation of calcareous deposits has priority over the biofilm attachment under cathodic protection. Calcareous deposits can build up below the biofilm.

The specimen with pre-existing calcareous deposits is subjected to slight corrosion when it was emerged in seawater for four weeks without cathodic protection, which implies that the calcareous deposits can inhibit the corrosion of carbon steel for about four weeks in a real ocean environment.

## Figures and Tables

**Table 1:** Energy-dispersive X-ray spectroscopy (EDS) analysis of calcareous deposits formed on anode-coupled bare samples (a) and anode-coupled samples with pre-existing calcareous deposits (b) after exposed for 1 week in natural seawater.

Element (wt %) Samples	C	O	Na	Mg	Si	P	Cl	Ca
anode-coupled bare samples (a)	9.02	41.33	0.67	0.30	1.06	1.09	-	46.51
anode-coupled samples with pre-existing calcareous deposits (b)	10.70	54.39	1.04	0.48	0.67	1.08	0.12	31.50

**Table 2:** Electrochemical parameters obtained from polarization curves measurements at different exposure time. SCE: saturated calomel electrode.

Samples	Exposure Time (Week)	-b_c_ (mV/dec)	b_a_ (mV/dec)	R_p_ (Ω·cm^2^)	E_corr_ (mV/SCE)	i_corr_ (mA/cm^2^)
A	1	226	88	1193	−679	0.0231
	2	205	114	907	−778	0.0351
	4	277	282	1671	−839	0.0363
	8	288	232	1302	−879	0.0429
	20	206	311	216	−828	0.2491
B	1	87	183	6243	−826	0.0041
	2	57	59	5700	−905	0.0022
	4	157	269	2336	−889	0.0184
	8	376	245	3063	−926	0.0210
	20	177	228	4834	−929	0.0090
C	1	131	172	1612	−803	0.0200
	2	157	224	1988	−800	0.0202
	4	314	181	1560	−804	0.0320
	8	310	238	1268	−861	0.0461
	20	139	224	199	−880	0.1872
D	1	132	235	5547	−1042	0.0066
	2	151	292	2529	−1043	0.0171
	4	249	473	2628	−903	0.0270
	8	153	183	4624	−956	0.0078
	20	225	295	5477	−925	0.0101

A, anode-uncoupled bare samples; B, anode-coupled bare samples; C, anode-uncoupled samples with pre-existing calcareous deposits; D, anode-coupled samples with pre-existing calcareous deposits; i_corr_, corrosion current density calculated by using Stern–Geary equation.
